# A Hybrid TOA-Fingerprinting Based Localization of Mobile Nodes Using UWB Signaling for Non Line-Of-Sight Conditions

**DOI:** 10.3390/s120811187

**Published:** 2012-08-10

**Authors:** Md. Humayun Kabir, Ryuji Kohno

**Affiliations:** Division of Physics, Electrical and Computer Engineering, Yokohama National University, 79-5 Tokiwadai, Hodogaya, Yokohama 240-8501, Japan; E-Mail: kohno@ynu.ac.jp

**Keywords:** UWB signaling, node localization, target tracking, fingerprinting, TOA, NLOS

## Abstract

Recently, Impulse Radio Ultra Wideband (IR-UWB) signaling has become popular for providing precise location accuracy for mobile and wireless sensor node localization in the indoor environment due to its large bandwidth and high time resolution while providing ultra-high transmission capacity. However, the Non-line-of-sight (NLOS) error mitigation has considerable importance in localization of wireless nodes. In order to mitigate NLOS errors in indoor localization this paper proposes and investigates a novel approach which creates a hybrid combination of channel impulse response (CIR)-based fingerprinting (FP) positioning and an iterative Time of Arrival (TOA) real time positioning method using Ultra Wideband (UWB) signaling. Besides, to reduce the calculation complexities in FP method, this paper also introduces a unique idea for the arrangement of reference nodes (or tags) to create a fingerprinting database. The simulation results confirm that the proposed hybrid method yields better positioning accuracies and is much more robust in NLOS error mitigation than TOA only and FP only and a conventional iterative positioning method.

## 1. Introduction

Lately, the Impulse Radio (IR)-Ultra Wideband (UWB) has been applied for the wireless personal area network (PAN), body area network (BAN), and RFID standards IEEE802.15.4a, 802.15.6, and 802.15.4f, respectively, because IR-UWB can perform ultra-high resolution of ranging, positioning, and high speed data transmission. Among the variety of prospective applications of IR-UWB, one of the most promising is in wireless sensor networks (WSNs), which require both robust communications and high-precision ranging capabilities [1]. Moreover, IR-UWB has some inherent properties like low power consumption, low cost, little interference to other systems, resistance to severe multipath conflicts and jamming, and has very high time domain resolution allowing for precise location and tracking, making it well suited for WSN applications [1].

Since IR-UWB signals have very short duration pulses, they can provide very accurate ranging and positioning capability in short range indoor radio propagation environments. Besides, the high time resolution characteristic of the UWB signal makes the time of arrival (TOA) method a good choice for location estimation in UWB communications. However, UWB localization and communication have to face lots of challenges like signal acquisition, multipath effects, multi-user interference and NLOS propagation. Among them NLOS propagation introduces positive bias for ranging estimation. Hence, it is difficult to recognize the impact of NLOS conditions on localization systems and to develop techniques that mitigate their effects [2].

Although, with enhanced sampling rates UWB receivers can accomplish high TOA resolution, their operating ranges are typically limited in reduced interference [3]. On the other hand, a suitable alternative to this real-time geometric positioning method is RF fingerprinting (FP) or pattern matching method, where a pre-calculated location database is used to match the properties of the received signal for position estimation. Moreover, the FP method is well suited in positioning under dense multipath conditions.

Besides the FP method, to achieve higher accuracy in localization of mobile nodes, many conventional iterative methods like Gauss-Newton, Steepest Descent, Levenberg-Marquardt have been proposed in conjunction with TOA or TDOA methods for mitigating NLOS errors. Among them, the Steepest Descent algorithm shows the slowest convergence in the final iteration steps, but for maintaining higher accuracy and low complexity the Levenberg-Marquardt method is the most suitable one to estimate a node location among the iterative methods [4]. Moreover, these iterative methods require initial positioning guesses. If the guesses are not accurate, convergence problems may occur. Alternately, a non-iterative positioning method can provide good initial positions for an iterative positioning method [5]. This idea also leads to an energy efficient iterative localization scheme for wireless sensor nodes. In [6], a hybrid combination of pattern matching (PM) and TDOA positioning methods is proposed for a CDMA network, although the described method has low accuracy in an indoor environment where the GPS signal is not satisfactory.

In our proposal, we introduce a hybrid positioning technique which combines a Channel Impulse Response (CIR)-based FP method with polygonal arrangement (which will be discussed in a later section) of reference nodes (or tags) and an iterative-TOA positioning method using UWB signaling for wireless *ad hoc* networks such as PAN, BAN, and RFID as well as wireless sensor nodes. The proposed method assures significant improvement in positioning accuracy compared to TOA only, FP only and a conventional iterative-TOA positioning methods by reducing NLOS errors effectively in the indoor environment. Moreover, we employ a deterministic wave propagation model based on Geometrical Optics to predict indoor radio coverage and CIRs.

In this paper, we would like to concentrate on the successful implementations of our proposed hybrid method in an indoor radio propagation environment such as a typical office room. We shall discuss on how to utilize the positioning results of the CIR-based FP method with different polygonal arrangements (which will be discussed in Section 3) of reference nodes in an iterative-TOA positioning method and to get the mutual benefits of the TOA and FP methods in a hybrid method. Besides, we would like to limit our discussion on the positioning performances of FP and proposed hybrid localization considering various NLOS incidences experienced by access points (AP)s. We also compare the performances of proposed hybrid method with TOA only, FP only and a conventional iterative-TOA method in LOS cases as well as worst cases of NLOS. Besides, the detailed mathematical analysis of computational complexity of the proposed hybrid method is beyond the scope of this paper and hopefully would be provided in a future issue.

The remaining of the paper is organized as follows: Section 2 illustrates the preparations for our proposal for hybrid positioning algorithm. The detailed analysis for our proposal for hybrid algorithm is provided in Section 3. Section 4 summarizes the model's performance using computer simulations. Finally, a brief conclusion is made in Section 5.

## 2. Preparations for Our Proposal for Hybrid Positioning Algorithm

Before introducing our hybrid positioning algorithm the following three subsections are the necessary preparations for our proposal. Section 2.1 discusses and proposes a deterministic channel model constructed by a Ray Tracing technique for indoor radio wave propagation. Then, the CIR-based FP positioning method for an indoor propagation environment is explained in Section 2.2. Section 2.3 provides an overview on UWB TOA-based accurate ranging in a multipath environment.

### 2.1. Deterministic Channel Model for Indoor Radio Wave Propagation

We consider a typical well-furnished office room that provides an efficient means of simulating a short range indoor UWB channel. Due to reflection, transmission, refraction, and diffraction by obstacles signal propagation suffers from severe multipath effects in an indoor environment [7]. In order to predict the accurate indoor radio coverage and channel impulse response we consider a deterministic (site-specific) propagation modeling technique, called Ray Tracing (RT). The RT technique utilizes the knowledge of the locations and electromagnetic properties of indoor objects, is used to predict path loss, time invariant impulse response, and the RMS delay spread [8–10].

A general model of band-limited complex channel impulse response (CIR) is expressed as [11]:

(1) $$h(t)=\sum_{n=1}^{N}A_{n}\delta (t-\tau_{n})e^{-j\theta_{n}}$$

where, *A_n_* is the path attenuation, *τ_n_* is the time delay of the *n*th path, *δ* is the Dirac delta function, *θ_n_* is the phase of the *n*th path. For the implementation of this model, it is necessary to identify the amplitudes, time delay and phases of the N number of components of the response.

**Figure 1.**
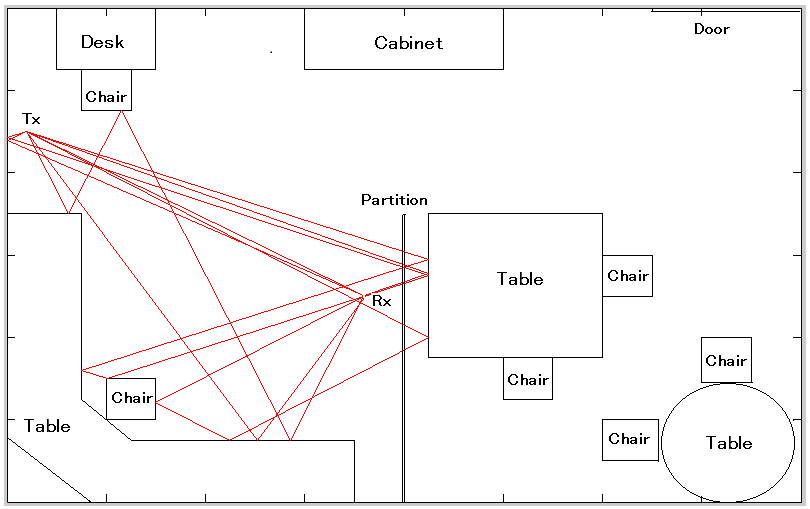
Ray tracing for indoor propagation modeling.

We employed the RT technique to identify the components of the above mentioned channel model. RT technique follows the ray launching approach (based on geometrical optics), which involves a number of rays launched uniformly in space around the transmitter antenna (Figure 1). Each ray is traced until it reaches the receiver or its amplitude falls below a specified limit. In our model, the specular reflections and transmissions are considered while diffraction and scattering are neglected, so that every wave component traverses one of more free space propagation paths between the transmitter and the receiver.

The complex electric field incident at the receiver due to the *n*th impinging ray can be represented as:

(2) $$E_{n}=E_{0}\frac{\lambda_{c}}{4\pi d_{n}}\left(\prod_{q=1}^{Q_{n}}P_{q}\prod_{r=1}^{R_{n}}\Gamma_{r}\right)e^{-j2\pi d_{n}/\lambda_{c}}$$

where:

*E*_0_: transmitted electric field
*λ_c_*: wave length correspond to center frequency
*d_n_*: propagation path length of *n*th ray
*R*_n_: number of reflections of *n*th ray
*Q*_n_: number of penetrations of *n*th ray
Γ_r_: coefficient of *r*th reflection of *n*th ray
P_q_: coefficient of *q*th penetration of *n*th ray

In Equation (2), the term $\frac{\lambda_{c}}{4\pi d_{n}}$ is the freespace pathloss, the exponential term $e^{-j2\pi d_{n}/\lambda_{c}}$ represents phase offsets due to pathlength *d_n_*, assuming omni-directional antennas where the azimuthal-plane antenna gains have been ignored for simplicity.

### 2.2. CIR Based FP Positioning Technique for Indoor Environment

The estimated CIRs described in the previous subsection are utilized for FP positioning and TOA based ranging (will be described in Section 2.3). The contents of Sections 2.2 and 2.3 are the preparations for the hybrid algorithm described in Section 3.

The CIR-based FP positioning, is also known as pattern matching technique, is especially suited for positioning under dense multipath conditions [12]. This technique consists of two phases:

1. In the training phase, location signatures (fingerprints) based on the received signal's CIRs' are measured by several access points (APs) for various reference locations of nodes in a region to construct a fingerprinting database.
2. In the positioning phase, the run time estimation of node's location is performed by correlating the CIRs' of received signal with the fingerprints stored in the database.

Generally, the FP technique requires exhaustive calculations for fingerprinting pattern matching as well as creating a database for storing the location fingerprints (signatures). Therefore, the computational complexity in the FP technique depends greatly on the size of the fingerprinting database employed in pattern matching. As the fingerprints of the reference nodes constitute a database, the larger the number of reference nodes the larger the size of the fingerprinting database needed to cover the whole area, but employing a smaller number of reference nodes reduces the computational complexity, although it will not provide a good accuracy in positioning. In order to combat this positioning constraint we introduce a new idea to optimize the number of reference nodes to cover the whole area as well as to generate a fingerprinting database of optimum size.

**Figure 2.**
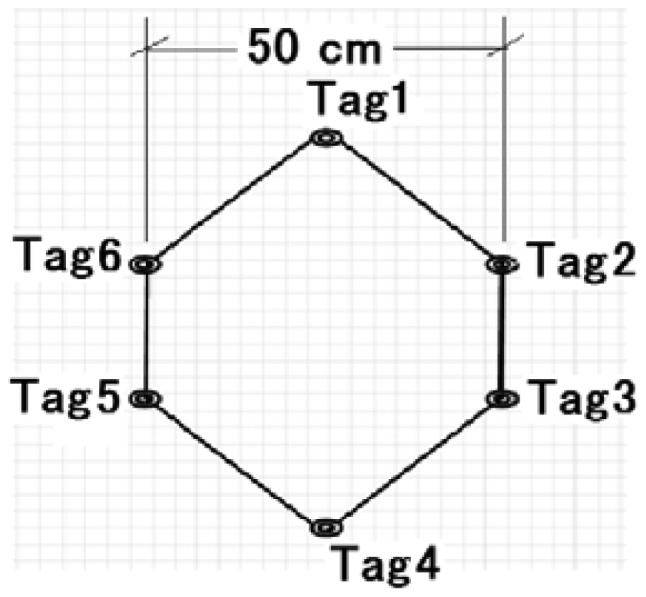
Size of polygon.

**Figure 3.**
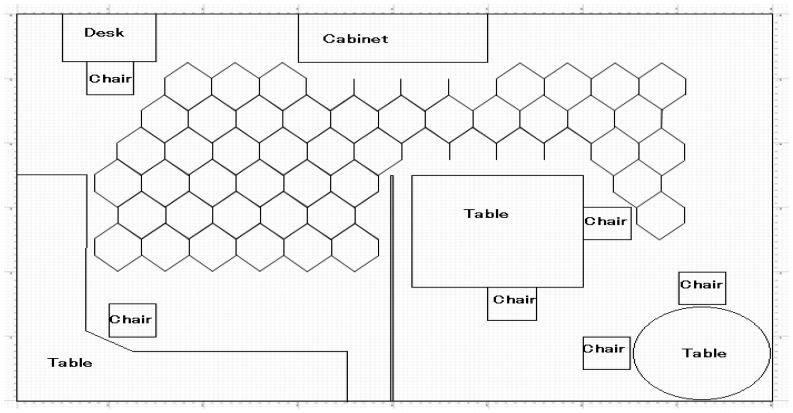
Arrangements of reference nodes in the room forming polygonal shapes.

In our new idea, we arrange the reference nodes in the room to form polygon shapes (as shown in Figures 2 and 3) instead of deploying them in a scattered way. The number of reference nodes required to cover the whole area depends on the size and quantity of such polygons. Similarly, the size of fingerprinting database also depends on the size of the polygons. Therefore, the optimum polygon size will reduce the computational complexity in fingerprinting pattern matching to a reasonable level while maintaining a certain degree of positioning accuracy in the FP method, so the main idea of arranging reference nodes (or tags) to form polygons is to reduce unnecessary deployment of reference nodes which increase the size of the fingerprinting database. Moreover, by varying the size of such polygons we can vary the size of the fingerprinting database as well as the complexity of the FP method calculations.

In the training phase of the FP method, signatures (fingerprints) h(l,t) are estimated and recorded for various reference locations I ∈ R, where R is the region of interest. Location of a node (or, tag) in 2D horizontal plane is defined as l(x,y) in Cartesian coordinates.

In the positioning phase, an estimate ***l̂***_0_ of the true instantaneous position ***l***_0_, is obtained by using the corresponding instantaneous estimated CIR, *h***(*l̂***_0_,*t*). This estimated CIR is corrupted by channel noise.

For, *m* = *h*(***l***, *t*) and *n* = *h*(***l̂***_0_,*t*), the channel spatial correlation $R_{l}^{m,n}$ is defined as:

(3) $$R_{l}^{m,n}=\frac{E\{mn\}-E\{m\}E\{n\}}{\sqrt{\left[(E\{{\left|m\right|}^{2}\}-{\left|E\{m\}\right|}^{2})(E\{{\left|n\right|}^{2}\}-{\left|E\{n\}\right|}^{2})\right]}}$$

where *E*{. } denotes the expectation operator. The position of the node is estimated by maximizing the correlation coefficient $R_{l}^{m,n}$, i.e.,

(4) $${\hat{l}}_{0}=\arg\underset{l\in R}{\max}\left|R_{l}^{m,n}\right|$$

Due to imperfect channel estimation at the receiver the ideal correlation cannot be attained. Hence, a correlation threshold *R_th_* is used such that, $R_{l}^{m,n}\geq R_{th}$. The values of $R_{l}^{m,n}$ crosses *R_th_* are defined as $R_{l}^{m,n}=[R_{l1},R_{l2},\ldots ‮‮,R_{lk}]$, where *k* is the number of values of $R_{l}^{m,n}$ crosses *R_th_* Then the values of $R_{l}^{m,n}$ are indexed with their corresponding location coordinates in descending order. Finally, the node location is calculated by taking a weighted average of the coordinates of the first three locations in the index, assuming these three locations are very close to the node location.

### 2.3. UWB TOA Based Ranging in Multipath Propagation Environment

The TOA estimation technique used with UWB transmission can be used for conducting accurate ranging in indoor multipath environments. For the indoor multipath channel, the impulse response is usually defined as [13]:

(5) $$h(\tau )=\sum_{k=1}^{L_{p}}\alpha_{k}e^{j\phi_{k}}\delta (\tau -\tau_{k})$$

where *L_p_* is the number of multipath components and *α_k_*, *Φ_k_* and *τ_k_* are the amplitude, phase and propagation delay of the *k*th path, respectively. The range measurement between a transmitter and a receiver is typically corrupted by multipath fading, thermal noise, blockage of direct path (*i.e.*, NLOS path) and direct path excess delay [14].

For TOA estimation, we employ a threshold-based first path detection method which utilizes the strongest path of CIR. This method makes use of an iterative search back algorithm, referred in [14], to calculate the noise floor to be used in the detection of the first path.

## 3. Proposal for a Hybrid Localization Algorithm

### 3.1. A Brief Idea of the Proposal for Hybrid Localization

The deterministic channel model described in Section 2.1, the FP positioning method explained in Section 2.2, and followed by UWB TOA-based ranging technique described in the Section 2.3, are the preparation for the hybrid localization that will be described in this section. Our proposal for hybrid localization is a combination of the CIR-based FP positioning and iterative-TOA positioning methods.

**Figure 5.**
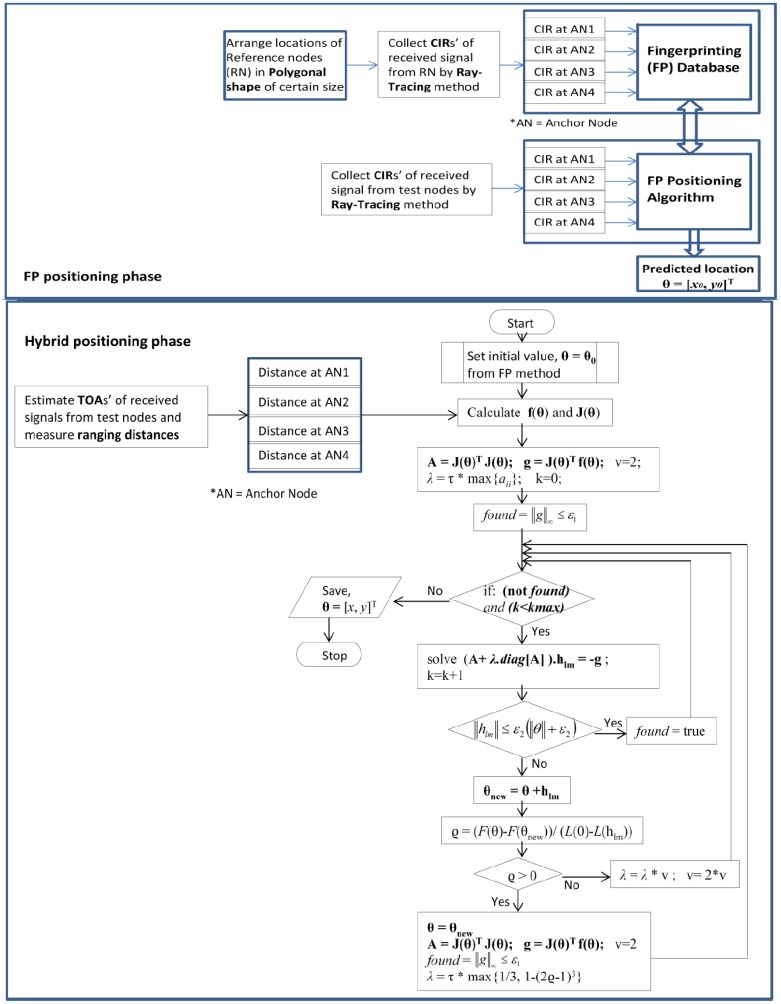
Flow chart for the proposed positioning algorithm.

It is obvious that an iterative positioning algorithm needs an initial guess which will influence the iterative process. A good approximation of the initial value also reduces the calculation complexity as well as increases the positioning accuracies. In our proposed method, we initialize the iterative-TOA positioning with position location estimated by the CIR-based precalculated FP method. As the CIR- based FP method has better positioning accuracy than the TOA method in dense multipath indoor conditions, the location point estimated in of the FP method acts as a better initial value for our hybrid method. Moreover, by varying the size of the fingerprinting polygons (as discussed in the previous section), the positioning errors as well the complexities in the proposed hybrid method could be minimized. The brief idea of the proposed localization algorithm is provided in the flow chart in Figure 5.

### 3.2. Hybrid Positioning Algorithm Combining CIR Based FP and Iterative-TOA Positioning Methods

In hybrid positioning, the node location estimated in the FP method is considered as the accurate initial values for an iterative-TOA method. The Levenberg-Marquardt based iterative algorithm (will be described in the later part of this section) is used to mitigate NLOS error in the iterative process of the hybrid positioning.

Let *M* access points (AP)s with position coordinates (*x_i_*, *y_i_*), where *i* = 1, 2,..,*M*. Assume (*x_0_*, *y_0_*) is the sensor node location to be determined and it is away from access points (AP)s. In the NLOS case, the measured range ***R****_i_* is always larger than the LOS range. This inequality can be expressed as:

(6) $$\text{LOS range}=\sqrt{{\left(x_{0}-x_{i}\right)}^{2}+{\left(y_{0}-y_{i}\right)}^{2}}\leq {\text{R}}_{i,i=1,2,\ldots ,M}$$

Let us define the range between position (*x*, *y*) and the AP*_i_* in LOS condition as:

(7) $$d_{i}(x,y)=\sqrt{{\left(x-x_{i}\right)}^{2}+{\left(y-y_{i}\right)}^{2}}{,}_{i=1,2,\ldots ,M}$$

As *d_i_*(*x*, *y*) is a nonlinear function, the measurement of *d_i_*(*x*, *y*) is erroneous in accordance with the severity of nonlinearity. This ranging error will be minimum at the immediate vicinity of the minimum of the cost function [15]. The nonlinear model of ranging can be represented as an optimum interpolation between a Taylor series method and a gradient method.

If (*x_es_*, *y_es_*) is an estimated position of the true position of node, expanding the function *d_i_*(*x*, *y*) in a Taylor's series we get:

(8) $$d_{i}(x,y)\approx d_{i}(x_{\mathrm{es}},y_{\mathrm{es}})+{\frac{\partial d_{i}(x,y)}{\partial x}|}_{y=y_{\mathrm{es}}}^{x=x_{\mathrm{es}}}(x-x_{\mathrm{es}})+{\frac{\partial d_{i}(x,y)}{\partial x}|}_{y=y_{\mathrm{es}}}^{x=x_{\mathrm{es}}}(y-y_{\mathrm{es}})$$

In Equation (8), only the terms of zero-order and first-order are kept. Let ***θ***_0_ = [*x*_0_, *y*_0_]^T^ be the position vector and **D**(***θ***_0_) = [*d*_1_(***θ***_0_), *d*_2_(***θ***_0_),…, *d*_M_(***θ***_0_)]^T^ be the distance vectors. According to Equation (8), the distance vectors **D**(***θ***_0_) can be written as:

(9) $$D(\theta_{0})\approx D(\theta_{\mathrm{es}})+J(\theta_{\mathrm{es}})(\theta_{0}-\theta_{\mathrm{es}})$$

where, the Jacobian matrix **J**(***θ****_es_*) can be expressed as:

(10) $$J(\theta_{\mathrm{es}})=\left[\begin{array}{cc} \frac{x_{\mathrm{es}}-x_{1}}{d_{1}(\theta_{\mathrm{es}})} & \frac{y_{\mathrm{es}}-y_{1}}{d_{1}(\theta_{\mathrm{es}})}\\ \ldots \ldots \ldots . & \ldots \ldots \ldots .\\ \frac{x_{\mathrm{es}}-x_{M}}{d_{M}(\theta_{\mathrm{es}})} & \frac{y_{\mathrm{es}}-y_{M}}{d_{M}(\theta_{\mathrm{es}})} \end{array}\right]$$

Thus, the most *M* linear independent ranges are:

(11) $$D(\theta )={\left[{\text{D}}_{1}(\theta ),{\text{D}}_{2}(\theta ),\ldots {\text{D}}_{\text{M}}(\theta )\right]}^{\text{T}}$$

and the corresponding observed ranges are given by:

(12) $$R=[R_{1},R_{2},\ldots ..R_{M}]$$

Then, according to [16,17] the cost function is defined as:

(13) $$\Phi (\theta )=\frac{1}{2}\sum_{i=0}^{M}{\left[R_{i}-D_{i}(\theta )\right]}^{2}$$

which should be minimized for the optimum case.

The minimization of tshe cost function is shown as:

(14) $$\hat{\theta }=\arg\min\Phi (\theta )$$

Then, we assemble the individual components of [*R*_i_ *–D*_i_(*θ*)] from Equation (13) into a residual vector ***ε***: R^n^→R^m^ defined by:

(15) $$\varepsilon (\theta )={\left[\varepsilon_{1}(\theta ),\varepsilon_{2}(\theta ),\ldots \varepsilon_{M}(\theta )\right]}^{T}$$

Using the notation ‖. ‖_2_ for Euclidean norm, we can rewrite Equation (13) as:

(16) $$\Phi (\theta )=\overset{1}{\;}/\underset{2}{\;}{\left\|\varepsilon \right\|}_{2}^{2}$$

Consider the linear case, where every *ε*_i_(*θ*), *i* = *1*,*2*,*…,M* is linear and Jacobian *J* of ***ε*** is constant. Then the cost function Φ is given by the quadratic as [16,17]:

(17) $$\Phi (\theta )=\overset{1}{\;}/\underset{2}{\;}{\left\|J.\theta +\varepsilon \right\|}_{2}^{2}$$

(18) $$\nabla \Phi (\theta )=J^{\text{T}}(J.\theta +\varepsilon )$$

(19) $$\nabla^{2}\Phi (\theta )=J^{\text{T}}J$$

Setting Φ(*θ*) = 0 yields *θ*_min_ = − (*J*^T^ *J*)^−1^ *J*^T^***ε***, which is the solution to the set of normal equations. For the non-linear case, as mentioned in [16,17],we have:

(20) $$\nabla \Phi (\theta )=J^{\text{T}}\varepsilon$$

(21) $$\nabla^{2}\Phi (\theta )=J^{\text{T}}J$$

In order to estimate the node position by reducing NLOS error, it is required to minimize the cost function Φ(*θ*). We applied the Levenberg-Marquardt [15,17] iterative algorithm that minimizes Φ(*θ*) and can be expressed as:

(22) $$\begin{array}{c} \theta^{\text{n}+1}=\theta^{n}+{\left(J^{\text{T}}J+\lambda^{\text{k}}\text{diag}[J^{\text{T}}J]\right)}^{-1}.J^{\text{T}}(\theta^{n})\varepsilon^{n}\\ =\theta^{n}+{\left(H^{k}+\lambda^{k}\text{diag}(H^{k})\right)}^{-1}g^{k} \end{array}$$

The Levenberg-Marquardt algorithm starts with an initial value, *θ*^(0)^ = [x^(0)^,y^(0)^]^T^ which is obtained from the FP method. In Equation (22), *λ* is the damping factor; whose value is reconfigured during iteration in order to reduce the influence of gradient descent. The value of *λ* affects both direction and step-size of iterations. Therefore, the initial value of *λ* should be chosen according to the size of the elements in *A^0^* = *J*(*θ^0^*)*^T^ J*(*θ^0^*), such that [18]:

(23) $$\lambda_{0}=\tau .max_{i}\{{a_{ii}}^{\left(0\right)}\}$$

Here, *τ* is defined by the user (by a rule of thumb, but a small value should be chosen). Throughout iteration the size of *λ* is updated and is controlled by the cost function Φ(*θ*). In an iteration, a smaller value of Φ(*θ*) indicates a good approximation to *θ ^n^*^+^*^1^*, and we can keep the weights at their new values and decrease *λ* by a certain (say, 10) factor so that the next Levenberg-Marquardt step is closer to the Gauss-Newton step. In contrast, if Φ(*θ*) is large, then *θ ^n^*^+^*^1^* is a poor approximation, and we should reset the weights at their previous values and increase *λ* by same factor for getting closer to the direction of Steepest Descent and reduce the steps.

## 4. Simulation Models and Performance Evaluations of Proposed Hybrid Method

This section provides the performances of our proposed hybrid method for node positioning by computer simulations. The performances of FP positioning method with various fingerprinting polygons (discussed in Section 2.2) are also evaluated in this section. Moreover, a comparison of the proposed hybrid method with TOA only, FP only and a conventional iterative method is shown in the later part of the section.

Tables 1 and 2 show the parameter values for evaluating model's performances and the properties of materials we employed in the RT technique, respectively.

To get the accurate results in the ray launching type RT technique, many rays have to be launched and only a fraction of these reach the receiver. The accuracy of this technique also depends on the radius of the reception sphere. If it is too small, rays will pass by. If it is too large, paths might be duplicated. We set the radius of the reception sphere at 1.5 cm. Moreover, we consider the reflection of rays up to the third order, since the received signal strength beyond third order ray reflection becomes very low and is neglected. CIRs collected by the RT technique are convoluted with a UWB signal with bandwidth of 2 GHz. To consider a sufficient amount of multipath effects in the CIR pattern for NLOS incidences we allow a wider observation window, *i.e.*, 65 ns, since the probability that the direct path is further apart from the strongest path is higher in the NLOS than in LOS. Therefore, it could accommodate maximum of 20 paths in the CIR patterns of NLOS cases.

As we consider an indoor office room of size 8 m × 6 m for our simulations, we think four APs are enough to cover radio mapping of this limited area and adequate for CIR-based FP positioning. It is obvious that increasing the number of AP would increase the possibility of LOS incidences and thus improve the accuracy in TOA-based positioning. However, increasing the number of APs will also increase the size of the fingerprinting database and thus increase the computational complexity of positioning in the FP method.

**Figure 6.**
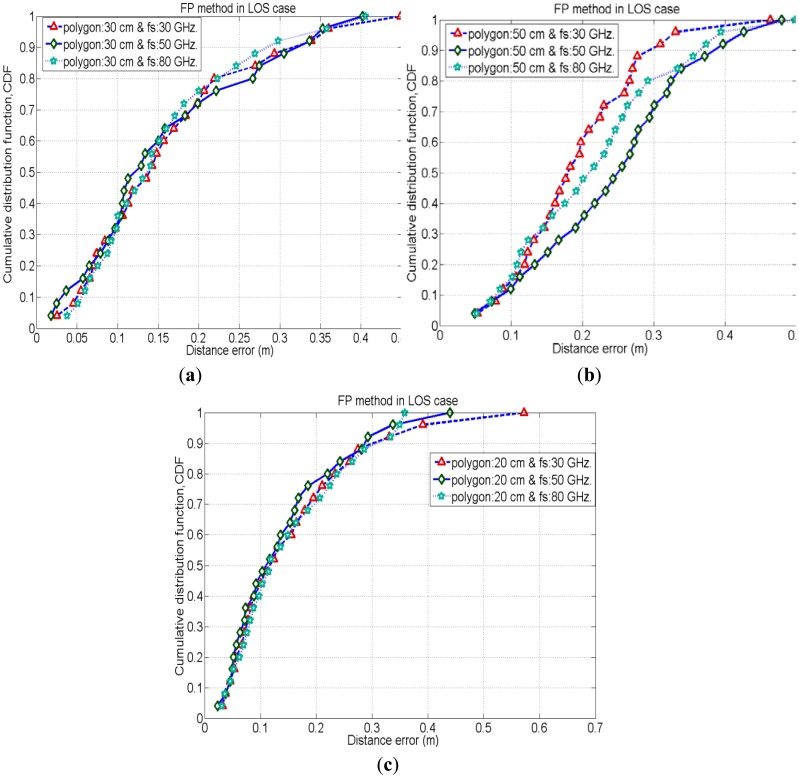
CDF of the distance errors for FP method in different cases with LOS.

**Figure 7.**
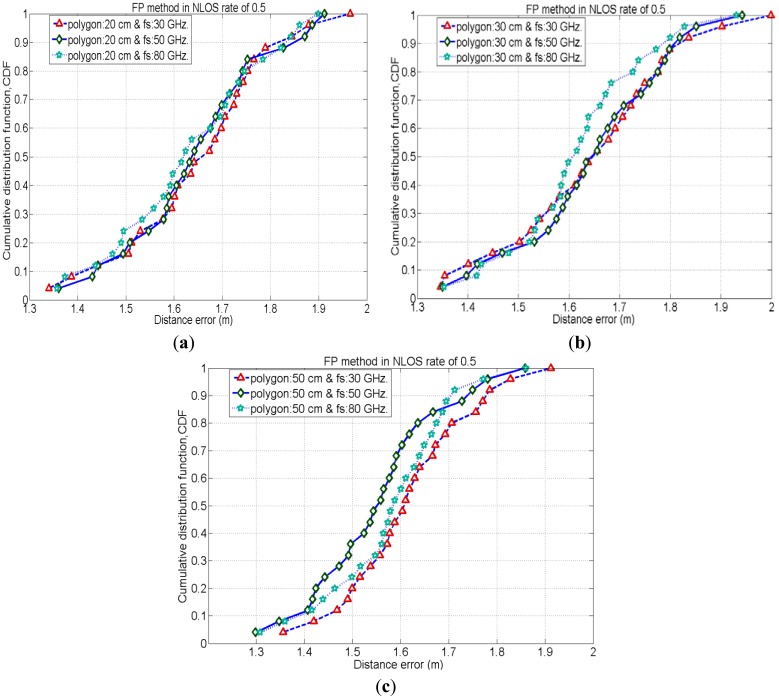
CDF of the distance errors for FP method in different cases with NLOS rate = 0.5.

**Figure 8.**
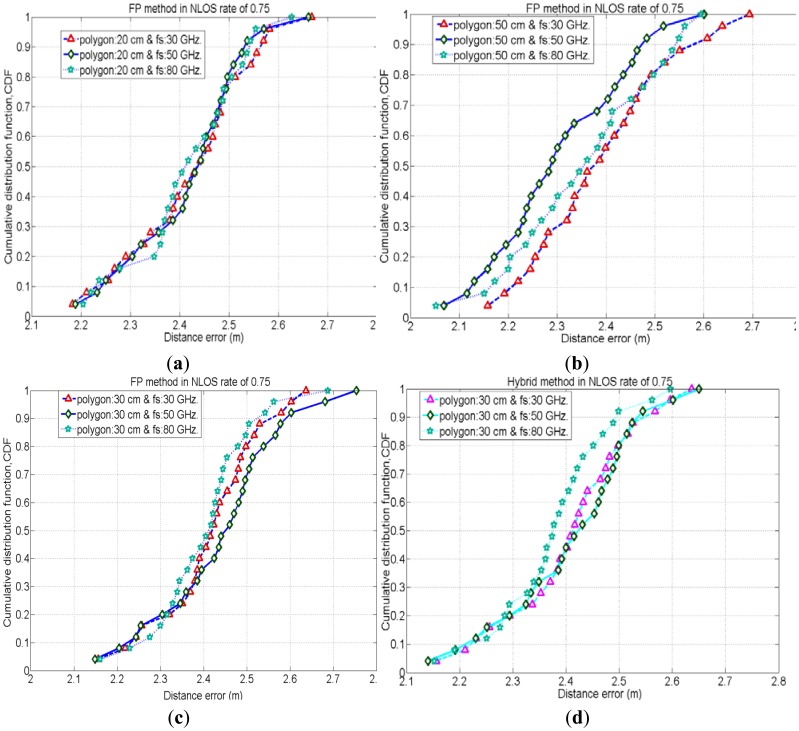
CDF of the distance errors for FP method (8**a**-**c**), Hybrid method (8**d**) in different cases with NLOS rate = 0.75.

To evaluate the performance of the FP method for positioning of the same test nodes which are used in an iterative TOA positioning, we place the reference nodes (or tags) in such a manner to form polygon shapes (as mentioned in Section 2.2). We employ three different sizes of polygon, 20 cm, 30 cm and 50 cm and evaluate the positioning accuracy by varying signal's sampling rate (*i.e.*, 30 GHz, 50 GHz and 80 GHz) for each case. Here, Figures 6–8 show cumulative distribution function (CDF) of distance errors (distance between estimated and real positions of test nodes) in the FP method for LOS cases as well as worst cases of NLOS. The NLOS rates shown in the figures are the ratios of the number of access points (AP)s experiencing NLOS among all APs. For instance, a NLOS rate of 0.5 means the half of the total number of APs are experiencing NLOS.

Figure 6 depicts that, in LOS cases, 50% probability is achieved for the accuracy of less than 10 cm in cases with a polygon size of 20 cm, whereas, 35% and 15% probability is achieved for the cases with polygon size of 30 cm and 50 cm, respectively, for the accuracy of less than 10 cm. Hence, cases with polygon size of 20 cm have better positioning accuracies than other polygon cases in LOS. This is because in cases with smaller polygons the reference nodes (or tags) are in close proximity to the test nodes positions as compared to the cases with larger polygons. Therefore, in smaller polygons, there are fewer chances of errors in matching the CIR pattern of the test node with that of its close neighbouring reference nodes under LOS conditions.

In NLOS situations, unlike LOS conditions the position estimation mostly depends on the multipath components of the received signal. Therefore, the estimated position would be very far away from the real position of the test node in NLOS conditions. As the estimated position is shifted from its real position, it is more likely to match the CIR pattern of estimated location with that of a far location point rather than matching CIR pattern of the point near to its real position. Figures (7) and (8) show the CDF of distance error in the FP method for worst cases of NLOS. With a NLOS rate of 0.5, the case with polygon size of 50 cm (with sampling rate 50 GHz.) has a 35% probability to achieve accuracy of less than 1.5 m, whereas the cases with polygon sizes of 30 cm and 20 cm have a probability of 20% to achieve accuracy of less than 1.5 m. Therefore, the case with polygon size of 50 cm (with sampling rate 50 GHz.) performs slightly better than other cases with different polygons because when the NLOS rate is 0.5, two of the APs cannot see the test node and CIR patterns measured by these NLOS APs are also considered for node positioning. These two NLOS CIR patterns of the test node could have a better match with the CIR patterns of reference node points of a polygon size of 50 cm (which are relatively far away from the real position of the test node) rather than matching with the CIR patterns of reference node points of polygon size 30 cm and 20 cm. Similarly, when the NLOS rate is 0.75, three out of four APs cannot see the test node and CIR patterns measured by these three NLOS APs are also considered for node positioning. Therefore, in these worst NLOS conditions, for the reasons stated above, the case with polygon size of 50 cm performs slightly better than other cases.

Besides, in the above results, the cases with higher received signal sampling rate sometimes provides slightly better positioning accuracies. Because, the higher sampling rate of the received signal reduces the time quantization error in the time delay estimation. Therefore, positioning accuracy increases with higher time resolutions.

It is obvious that, the smaller the polygon size the larger the fingerprinting database as well as the higher calculation complexity will be in the FP method. Alternatively, the cases with the larger polygon size provide lesser calculation complexity in FP positioning.

**Figure 9.**
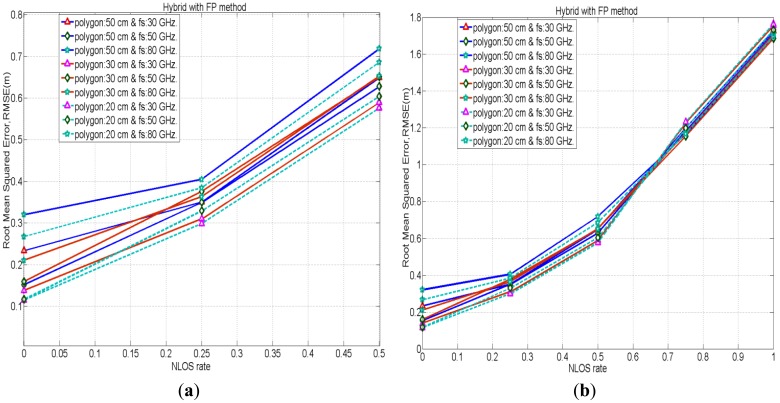
Positioning accuracies in hybrid method for various cases with polygon sizes in LOS and different NLOS conditions.

Figure 8(c,d) depicts the comparison of positioning accuracies in between FP and the proposed hybrid method for the cases with polygon size 30 cm when the NLOS rate is 0.75. Figures depict that the proposed hybrid method has better positioning accuracies by mitigating NLOS errors than the FP method. Figure 9 shows the performances of the hybrid positioning in root mean squared error (RMSE) for various cases with polygon sizes in LOS as well as different NLOS conditions. Moreover, the smaller polygon size in FP positioning provides better location accuracies in the hybrid method for lower rates of NLOS, whereas, for higher rates of NLOS, the positioning accuracies for the cases with various polygon sizes are almost identical.

In the proposed hybrid method, there is possibility to get mutual benefits from the TOA only and FP only methods. If any case in FP method does not have better positioning accuracy than the TOA only method, so the hybrid method can choose its initial value from the positioning result of TOA only method for iterations.

**Figure 10.**
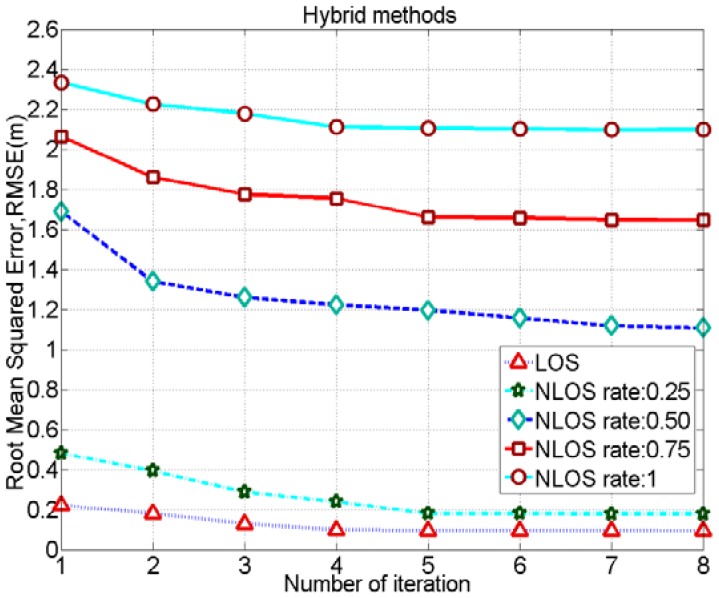
Performance of the hybrid method in each iteration for different NLOS conditions, considering fingerprinting polygon size: 20 cm and signal sampling rate: 50 GHz.

The positioning accuracies for different cases using the proposed hybrid method can be increased by increasing the iteration number. However, as depicted in Figure 10, the accuracy does not increase beyond 4 or 5 iterations for various NLOS cases. Hence, setting a maximum iteration number beyond 4 or 5 would not be necessary. Moreover, in Figure 10, there is a wide gap between the RMSE performances of NLOS rates of 0.25 and 0.5. This is because we have a better positioning accuracy in the FP method using a polygon size of 20 cm when the NLOS rate is 0.25. Therefore, in hybrid positioning, RMSE for the case of a NLOS rate of 0.25 decreases and it is closer to the positioning accuracy of the case with LOS where the polygon size is 20 cm. Besides, we don't have better positioning accuracy for the case using a polygon size of 20 cm than the case of polygon size of 50 cm in the FP method while NLOS rate is 0.5. Therefore, in hybrid positioning, RMSE performances in each iteration for the case of NLOS rate of 0.5 are not good enough. Moreover, it is obvious that if we increase the number of test node samples in the FP method the positioning accuracy for the case of NLOS rate of 0.5 would also increase a bit.

**Figure 11.**
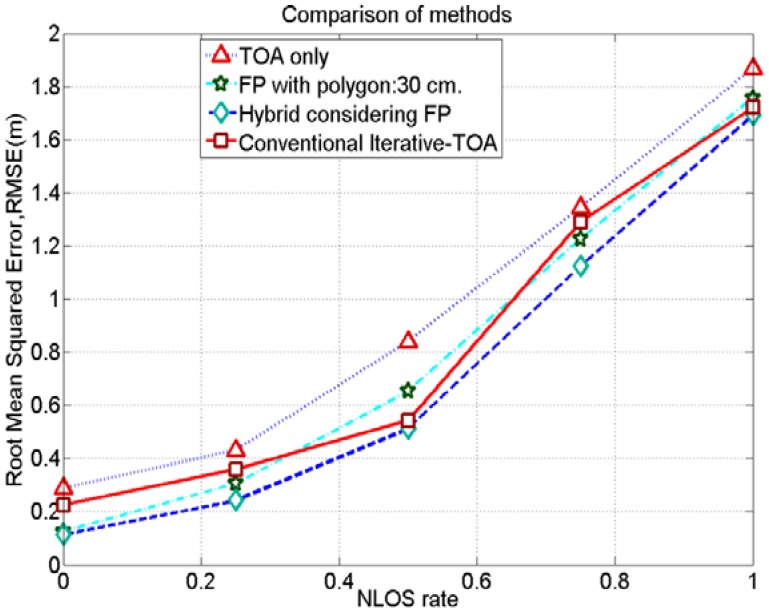
Comparison on positioning accuracies for TOA only, FP only, conventional iterative TOA and hybrid methods in NLOS conditions.

Figure 11 shows that the proposed hybrid method has better positioning accuracies by mitigating NLOS error than TOA only, FP only and a conventional iterative method under different NLOS conditions. In a conventional iterative TOA method, we also applied the Levenberg-Marquardt iteration algorithm; however, initial values are random guesses unlike considering the positioning results of the FP method. Similarly, the proposed method also renders better positioning accuracies than the Gauss-Newton based iterative-TOA positioning method, reported in [19].

## 5. Conclusion

We have proposed a hybrid approach for localization of mobile or wireless sensor nodes using UWB signaling which combines the CIR-based FP positioning and iterative-TOA real time positioning methods. The proposed hybrid method follows a Levenberg-Marquardt based iterative algorithm, which effectively reduces NLOS errors. Our simulation results show that the proposed hybrid method yields better performances and is more robust in NLOS error mitigation than TOA only, FP only and a conventional iterative method. Moreover, the smaller size of polygons, resembling reference nodes (or tags) arrangement in the FP method, the bigger the size of fingerprinting database as well as the higher calculation complexity in positioning. Alternately, the larger size of polygon provides a smaller fingerprinting database compromising positioning accuracies. The optimum size of polygon could be predicted while evaluating the trade-off between accuracies in the proposed hybrid method and the complexities in positioning calculations, which is beyond the scope of this paper. A detailed analysis of the computational complexity of the proposed method will be provided in our next paper on this topic.

## Figures and Tables

**Figure 4.**
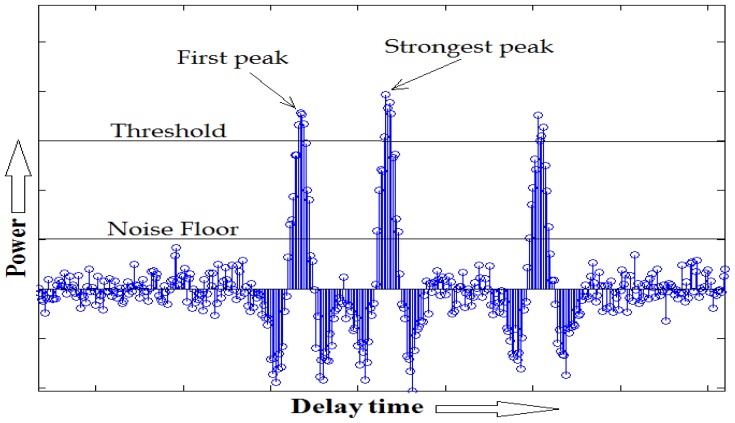
Search-back detection method.

**Table 1.** Parameter values for evaluating model's performance.

**Parameter**	**Value**
Transmitted signal	Gaussian monopulse
Channel bandwidth	2 GHz
Sampling frequencies	30, 50, 80 GHz.
AWGN parameter	σ = 0.5 m
Transmitted Power	−60 dBm
Fingerprinting polygon size	20 cm, 30 cm, 50 cm
Number of Access Point	4
Area	8 m × 6 m

**Table 2.** Properties of the materials employed in RT technique.

**Materials**	**σ** [**S/m**]	**ε_r_**	**Thickness**[**cm**]
Concrete wall	0.01	9	7.5
Table, chair, cabinet (wood)	10^−5^	13	3.0
Window (glass)	10^−12^	7.6	3.0
